# Deflection of sliding droplets by dielectrophoresis force on a superhydrophobic surface

**DOI:** 10.1038/s41598-024-62925-z

**Published:** 2024-05-30

**Authors:** Yun-Han Bai, Shih-Yuan Chiu, Hong-Ren Jiang

**Affiliations:** 1 Institute of Applied Mechanics, National Taiwan University, No.1, Sec. 4, Roosevelt Rd., Da’an Dist., Taipei City, 106 Taiwan (R.O.C.); 2 Department of Electrical Engineering, National Taiwan University, No.1, Sec. 4, Roosevelt Rd., Da’an Dist., Taipei City, 106 Taiwan (R.O.C.)

**Keywords:** Applied physics, Techniques and instrumentation

## Abstract

In this study, we experimentally identify the effect of liquid dielectrophoresis (LDEP) force on a superhydrophobic surface in directing the trajectory of moving water droplets across designed interdigitated electrodes and show that this method is capable of rapidly selecting droplets at a high speed (200 mm/s). As the droplets traverse down the surface by the electric field, their deflection on the edge of these electrodes is achieved successively, allowing for the selective manipulation of discrete droplets. A series of experiments were conducted to validate the relationships among droplet deflections, applied electric fields, and dynamic contact angles. Our findings reveal that the principal driving force behind the droplet deflections is the LDEP force, which can provide instant manipulation of moving droplets rather than a variation in contact angles brought about by electrowetting. This study presents a proof-of-concept experiment utilizing LDEP for high-throughput droplet selection and also highlights the potential applications of this mechanism in high-speed digital microfluidics (DMF) and biological separation methodologies.

## Introduction

Wettability denotes the capacity of a liquid to maintain contact with a solid surface, while electrowetting involves altering the liquid's wettability by applying an electric field^1^. Previous research^2–4^ has illustrated that surface tension can be modified in correspondence with the strength of the electric field, thereby enabling the manipulation of water droplet movement^5^. Moreover, properties of electrowetting on water droplets have been investigated on the hydrophobic surface, demonstrating the capability to modify droplets’ shape^6^ and induce oscillation of the contact angle^7^. Electrowetting effects have been extensively employed in digital microfluidics, serving purposes such as Lab-on-chip biological applications^8^, and electrowetting display (EWD) technologies^9–13^.

In contrast to the electrowetting effect, which utilizes a uniform electric field to modify the contact angle of a liquid surface, dielectrowetting employs a non-uniform electric field, generating a dielectrophoresis force through liquid polarization^14–16^. An advantage of dielectrowetting is its ability to apply higher voltage on water droplets with electrolytes, overcoming the constraint of contact angle saturation and electrolysis inherent in electrowetting^17,18^. Cutting-edge digital microfluidics have begun utilizing liquid dielectrophoresis to achieve dielectrowetting effects on platforms furnished with interdigitated electrodes^15,19–21^. Dielectrophoresis has also been explored to control droplets from splashing^22^ and to sort the droplet by bouncing^23,24^. However, the bouncing of droplets is sensitive to various factors like initial dropping speed and the incident angles, which may pose challenges in controlling the motion of droplets. Achieving precise control of droplets at high speeds continues to be a significant challenge.

In our study, we conducted experiments on superhydrophobic surfaces fitted with interdigitated electrodes to deflect moving droplets at high speeds. By manipulating electric fields, we demonstrated the ability to alter the adhesive force on droplets, thereby inducing their deflection. Our results indicate that the LDEP force can directly cause droplet deflections. The device setup features a straightforward design capable of handling high-speed droplet movements, potentially leading to applications in various fields, such as high-speed biological separation techniques and lab-on-a-chip devices for microfluidic actuation and manipulation.

## Results and discussion

**Figure 1:**
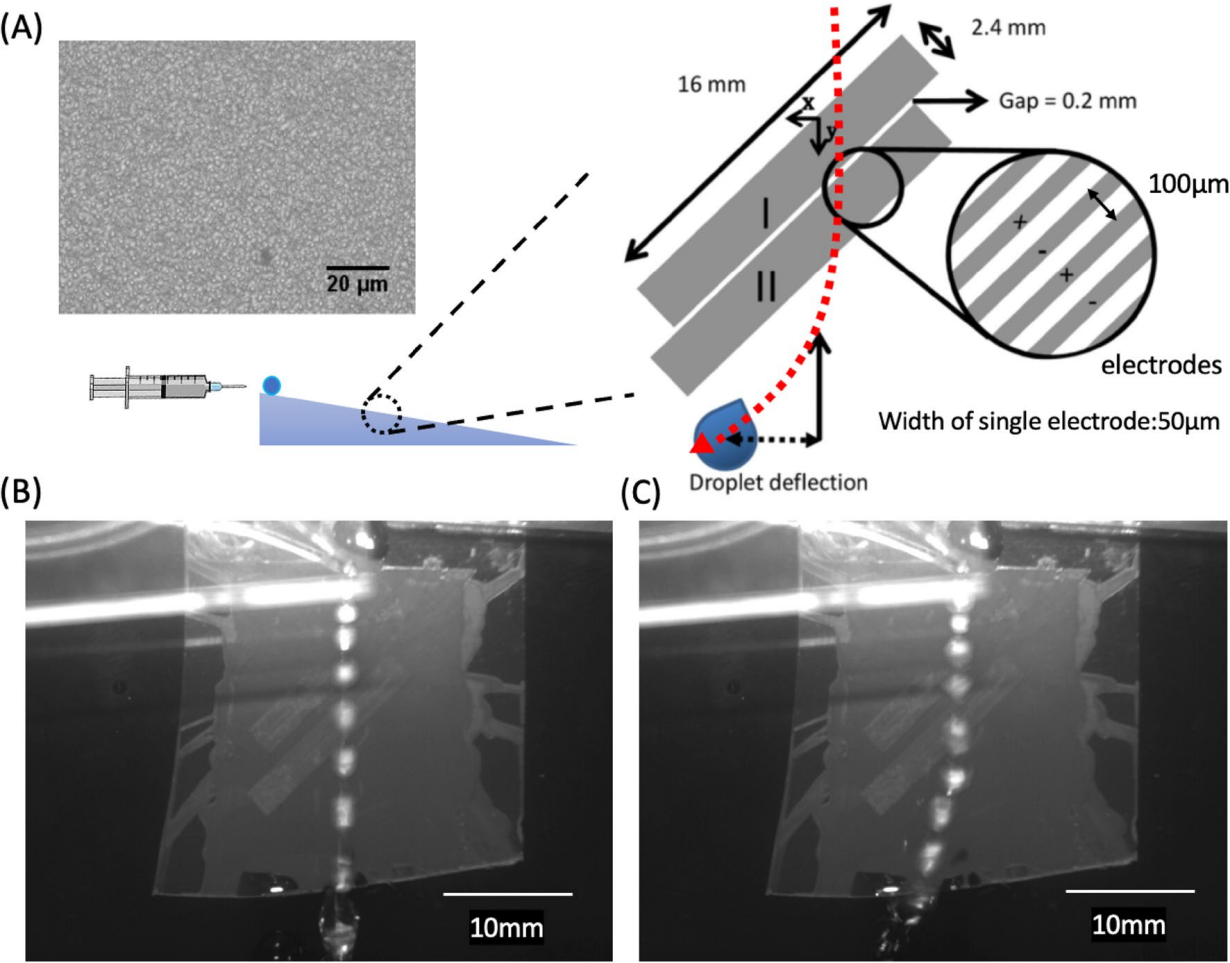
Deflection of droplets (**A**) Schematic diagram of the experimental setup of deflection of droplets. Inset image: superhydrophobic surface under optical microscope. (**B**) Trajectory of water droplets without voltage applied. To illustrate the droplet's position in each frame, the image was synthesized from ten frames at a frame rate of 60 (fps). (**C**) Trajectory of water droplets under 250Vrms.

The experiment investigating the deflection of deionized water droplets was conducted on a superhydrophobic interdigitated electrodes surface (see “Materials and methods” section). The schematic of the experimental setup was depicted in Fig. 1A. The interdigitated electrodes were oriented 45° from the vertical direction. A syringe pump was utilized to regulate the size of the water droplets as they slided down the surface. The sliding speed of the droplets could be managed by adjusting the tilting angle. The lateral displacements of a droplet were measured under varying voltages at fixed sliding velocities.

In the scenario where a droplet slided down the surface without any applied voltage, the trajectory of the water droplet is illustrated in Fig. 1B. The droplet maintained a straight path, exhibiting no deflection. However, at higher voltages, the droplet deflected at the edge of the electrode (II), with the trajectory of the water droplet at 250 Vrms (10 kHz) displayed in Fig. 1C (Supplementary Information). In order to prevent electrohydrodynamics in the liquid, we selected 10 kHz as our operating frequency. Droplets could be observed sliding down along the electrode (II).

**Figure 2:**
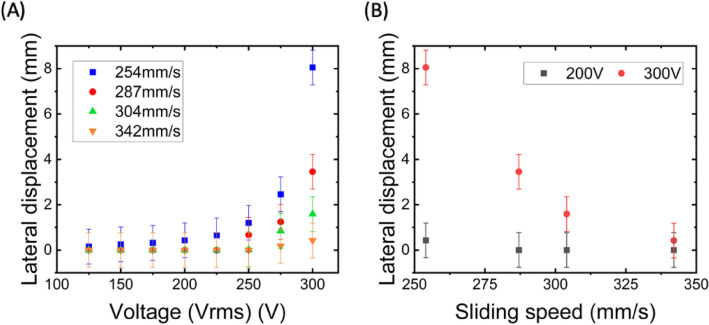
(**A**) The relation of voltages versus lateral displacement of water droplets under different sliding speeds. The error was estimated as ± 0.8 mm. (**B**) The relation of sliding speeds and lateral displacement of water droplets under 200 V and 300 V.

By applying varying voltages under different sliding speeds, the lateral displacements of droplets are documented, as demonstrated in Fig. 2A. Experimental errors incorporated the slight variations for the trajectory of sliding droplets. The positioning of droplets on the analysis software contributed to measurement errors. Four sets of data were measured, and the error was calculated to be ± 0.8 mm. In comparison to the droplets' diameter of 3 mm, the displacement of droplets is minimal at low voltages below 200 V, but the displacement enlarges with increasing voltage. Figure 2B showcases the lateral displacement versus different sliding speeds under the voltages 200 V and 300 V, indicating a significant increase in deflection as the voltage changes from 200 to 300 V. The deflection is also sensitive to the sliding speed. At a voltage of 300 V and a droplet sliding speed of 250 mm/s, the displacement value is 8 mm. However, when the sliding speed elevates to 350 mm/s, the displacement diminishes to 0.2 mm, suggesting that the deflection is almost negligible if the droplet sliding speed exceeds 300 mm/s.

Our findings demonstrate that water droplets deflect on the superhydrophobic surface under the influence of electric fields. To analyze the other potential forces involved in this process, we consider the Bond number and Capillary number, which indicate the relative magnitude of gravitational forces, surface tension, and viscous drag. The Bond number, Bo = ρgL^2^/σ, is estimated to be 1.21, where ρ is the density of water, σ is the surface tension of water, and L is the diameter of the droplets (approximately 3 mm). The resulting Bo value is close to 1, suggesting that the influences of gravitational force and surface tension are almost equally significant^25^. The Capillary number, Ca = μV/σ, where μ is the dynamic viscosity of the liquid and V is the characteristic velocity, is relatively small, indicating that viscous drag may not significantly affect the process. Therefore, we primarily consider the effects of both gravity and surface tension of droplets under the effect of electric fields.

To decipher the mechanism behind this deflection, we explore several potential mechanisms. Previous studies have indicated that electric fields can alter the contact angle of a liquid^1^. As droplets traverse the edge of the electrodes, the contact surfaces of water are exposed to varying electric fields, potentially altering the contact angle. To determine if electric fields can induce sufficient changes in contact angles to generate additional force on droplets, we assessed the contact angles under various voltages and analyzed the response time for altering the contact angle.

**Figure 3:**
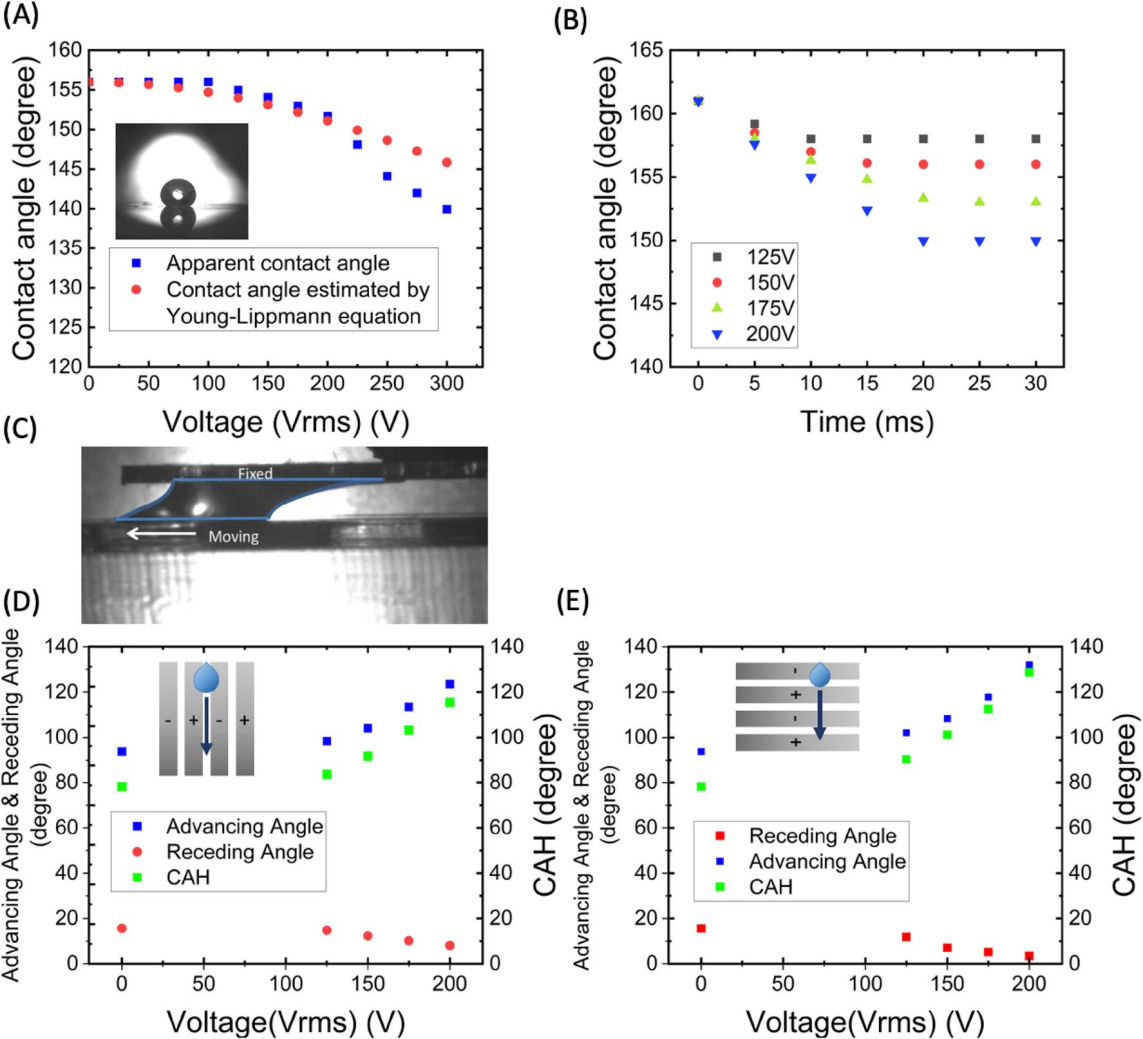
Contact angle and contact angle hysteresis under voltages and dynamic motion. (**A**) Blue line is the apparent contact angle under different voltages. Red line is the contact angles on the flat surface estimated by the LDEP form of Young-Lippmann Equation. Inset image: a water droplet at 0 V, where the apparent contact angle of the surface is 155*°*. (**B**) Contact angles responding time at different applied voltages. (**C**) Experimental setup of the motorized stage where the water droplet is placed between. To engender hydrophilicity on the upper slide glasses for pulling droplets, a piece of paper was adhered to the slide glass, followed by recording the dynamic contact angles. (**D**, **E**) With sliding speed 125 mm/s, the relation of CAH, advancing and receding angles versus voltages at two kinds of electrode orientation respectively.

Since the superhydrophobic surface contains nanoparticles, the contact angles measured are apparent contact angles in the study^26^. The experiment to measure static apparent contact angles was conducted. A water droplet was positioned flat on the surface of superhydrophobic interdigitated electrodes, and a 10 kHz AC electric field was applied to the surface. The correlation between the apparent contact angle and the applied voltage was evaluated at intervals of 25 V, increasing from 0 to 300 V as depicted in Fig. 3A. The data revealed that below 200 V, the alteration in apparent contact angle was minor, around 3°. At 300 V, the apparent contact angle was reduced by 15° in total. The red dots in Fig. 3A are contact angles estimated from the Young–Lippmann Equation:

1 $$\text{cos}\theta \mathcal{e}\left({\text{V}}\right)=\text{cos}\theta \mathcal{e}+\frac{\varepsilon o\left(\varepsilon \iota -1\right){\text{V}}^{2}}{2\times \gamma \iota \nu \times \delta }$$

where $\theta \mathcal{e}$ is the contact angle, $\varepsilon \iota$ is the permittivity of the liquid, $\delta$ is the penetration depth, $\gamma \iota \nu$ is the surface tension of the liquid–gas interface^14^. Penetration depth is related to the electrode structure by $\delta =\frac{4d}{\pi }$^14^, where d = 50 μm is the width of the device IDE. Although the Young-Lippmann Equation is derived for a flat surface, it may be worthwhile to compare it and see the difference.

Below 200 V, the experiment aligned with the contact angles estimated by Young-Lippmann Equation, whereas a larger deviation from the red line was evident at voltages exceeding 200 V. According to the results, the apparent contact angle shifted from 155° to 140° as the voltage increased from 0 to 300 V. The deviations may be attributed to the presence of nanoparticles on our surface, which are used to create its superhydrophobic properties. We also note that our surface exhibits better electrowetting properties at higher voltage. Additionally, a lag for water droplets to fully acclimate to the electric fields is measured to be roughly 20 ms, as demonstrated in the graph of apparent contact angle versus response time in Fig. 3B.

Utilizing these findings, we estimated the force exerted on the entire droplet due to the alteration in the contact angle. Given the surface tension of water is γ = 72 mN/m, and the diameter of droplets is 3 mm, the estimated force that caused the difference in contact angles was around 0.03 mN. While the force needed to deflect droplets was estimated to be approximately 0.08 mN, this magnitude of force was insufficient for droplet deflection. The time needed for a water droplet to stably alter its contact angle was 20 ms, while the transit time for water droplets to cross the interface was approximately 15 ms at 200 mm/s. Consequently, the water droplet rapidly traversed the electrodes, and its contact angle didn't fully adjust within this brief duration. The analysis of contact angle alterations and their response times implied that the changes in contact angles were not the only driving factor behind the deflection of droplets.

In exploring additional mechanisms, we turned our attention to a previously discussed phenomenon where the variance in contact angle hysteresis (CAH)^27–29^ could lead to the deflection of water droplets at the interface between two distinct surfaces^29–31^. To ascertain if CAH on different surfaces is the reason for deflection, we proceeded to measure the advancing angles, receding angles, and CAH in subsequent experiments under diverse electric fields and sliding velocities.

The experiment to measure the advancing angle, receding angle, and CAH was conducted on a motorized stage with IDE (see Materials and Methods). Water droplets were placed flat on the electrodes and with slide glasses on the top of the droplets in Fig. 3C. As the stage was moved at constant speeds of 100 mm/s, 125 mm/s, and 150 mm/s, we assessed the dynamic contact angles and CAH under varied AC voltages (10 kHz) of 125 V, 150 V, 175 V, and 200 V. The outcomes at 125 mm/s, with different electrode orientations, were illustrated in Fig. 3D and E, showcasing a marginal impact from the electrode directions on the experiment. With increasing applied voltage, the receding angle of the water droplet diminished from 15° to 10°, whereas the advancing angle surged from 90° to 130°. Consequently, the hysteresis effect intensified, and CAH climbed from 80° to 125° with the rising voltage.

This conjecture of droplet motion predicated on CAH measurements deviates from earlier study^30^, which posited that as water droplets transition from a high CAH to a low CAH on the superhydrophobic surface, the deflection force gravitates towards the low CAH surface. The deflection force was approximated as

2 $${\text{F}}\approx \gamma \iota \nu \times {\text{Ro}}\times \text{sin}\left(\pi -\theta \right)\times \left(\text{cos}\theta {\text{r1}}-\text{cos}\theta r2\right)$$

where $\gamma \iota \nu$ is the surface tension of the liquid–gas interface, Ro is the radius of the droplets, $\theta {\text{r1}}$ is the receding angle on the upper surface, $\theta {\text{r2}}$ is the receding angle on the lower surface^30,32,33^.

Contrastingly, our observations indicate that water droplets deflect along the high CAH surface as opposed to the low CAH surface, thereby suggesting that the alteration in CAH at the interface is not the determinant for the deflection of water droplets.

In addition to the previously discussed mechanisms (alteration in contact angle and CAH on diverse surfaces), we hypothesized that the water droplets encounter substantial resistance upon leaving the electrodes due to nonuniform electric fields. The droplets are polarized by the nonuniform electric field, generating a liquid dielectrophoretic (LDEP) force, which in turn, leads to the deflection of the water droplets. To comprehend the force acting on the water droplets, we undertook a force analysis during the sliding process on IDE (See Materials and methods).

**Figure 4:**
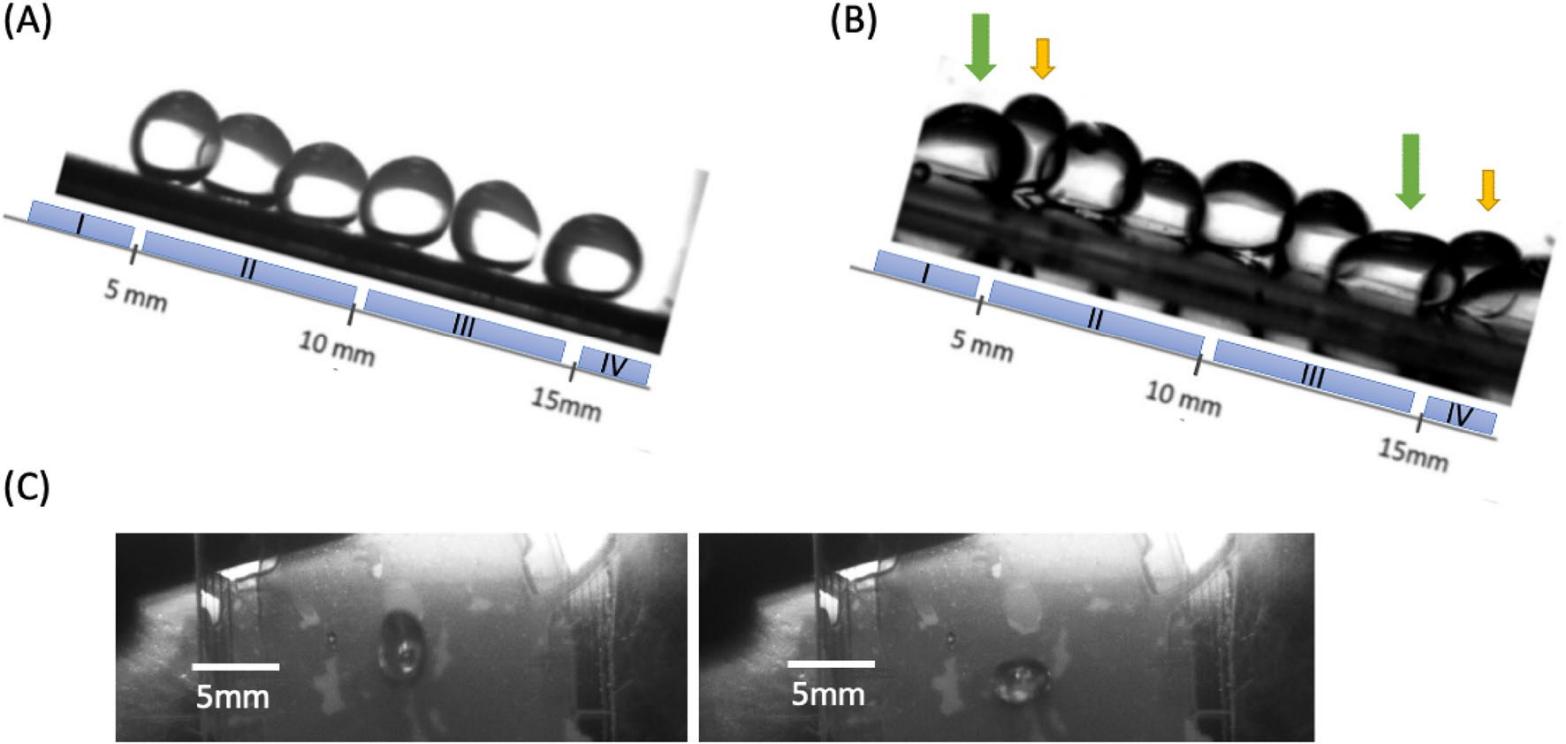
Shape of droplets in motion (**A**) Image of water droplets sliding down from interdigitated electrodes without electric fields applied. Water droplets are not perfectly round due to gravitational force, causing them to become flattened. Gravitational force has little impact on the contact angle, but it does affect the overall shape of the water droplets. (**B**) Image of water droplets with I, III electrodes turned on (200 V). Specific deformation can be observed on the edges of electrodes where the arrows point. Green arrows show the droplets are spread out on the surface (elongation state), while yellow ones show the droplets inclined to stay on the edges of I, III electrodes (compressed state). (**C**) Top view images for elongation state (left) and compressed state (right) of droplets.

The IDE units were designated I, II, III, and IV from top to bottom, as depicted in Fig. 4. As the droplet glided down from the top of the electrodes on a 15° inclined platform, the sliding progression was captured per 10 ms by a camera. With all electrodes deactivated, the droplet traversed down the surface as illustrated in Fig. 4A, maintaining its shape. Given that images of the droplet were captured at consistent time intervals, the amplification in the traveling distance of water droplets within equal time segments denoted an acceleration down the surface. With electrodes I and III activated, substantial deformation was noticeable in Fig. 4B. Green arrows elucidate that droplets expand toward the surface due to enhanced adhesion, while yellow arrows denote that the droplet is compacted in the longitudinal axis, enduring significant deformation at the interfaces of I, II and III, IV. The top view is shown in Fig. 4C.

**Figure 5:**
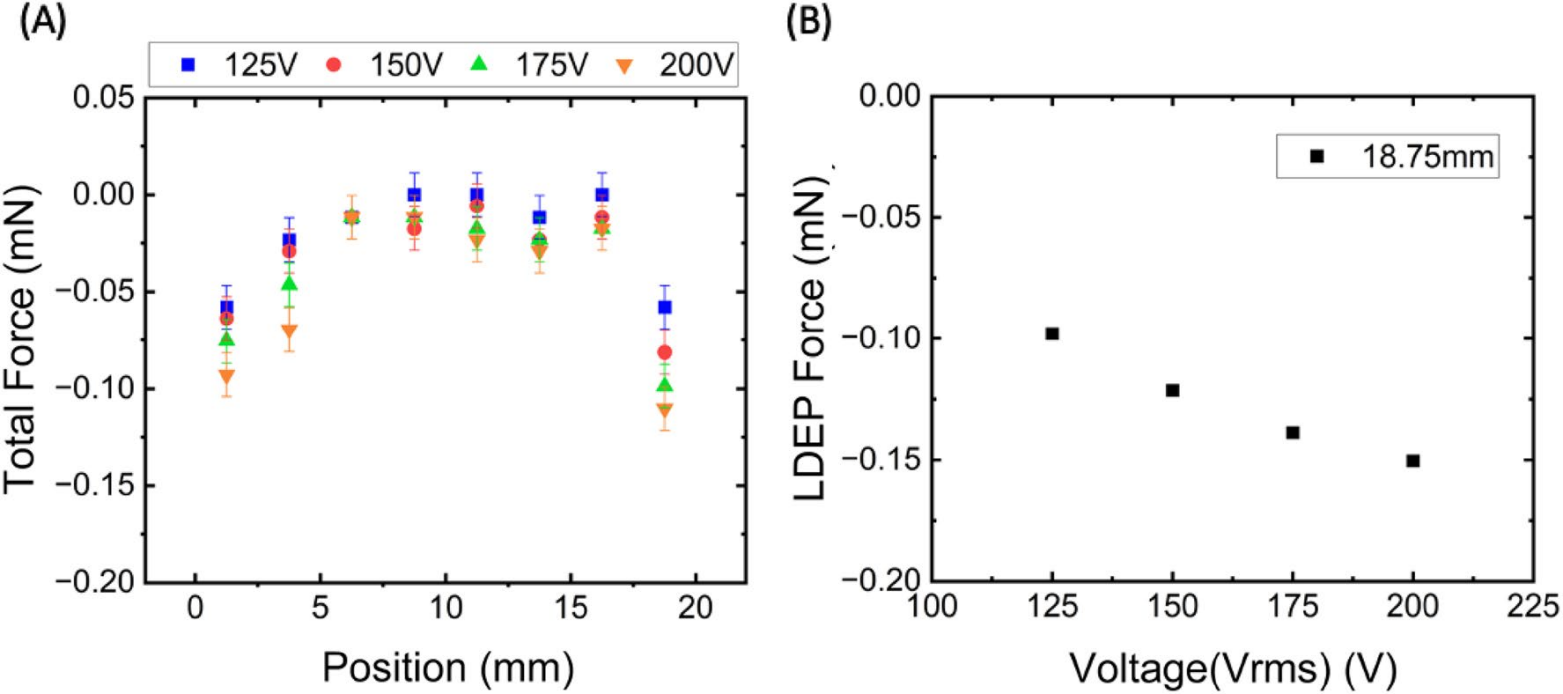
Analysis of dielectrophoresis force (**A**) With all the electrodes turned on, the estimated total force on the water droplets at different positions. (**B**) The relation of LDEP force versus the magnitude of voltage at the position of 18.75 mm on the electrodes.

To enhance the comprehension of the force influenced by electric fields on the droplet, we assessed the velocity of the droplet and the CAH during its sliding phase. By analyzing the velocity and the size of the droplets, we were able to ascertain the total force impacting the droplets with all the electrodes turned on, Fig. 5A. Four sets of data were measured, and the error was calculated to be ± 0.01 mN. From the CAH, we deduced the resistance attributed to hysteresis on the droplets.

Given that our experiment was conducted on a superhydrophobic surface, friction was disregarded, and therefore the resistance is contributed by LDEP force^14,17^. We approximated the gravitational force on droplets to be 0.04 mN towards the sliding direction on a 15° inclined surface. The sliding direction was designated as positive, hence a negative sign indicates a resisting force, directed upward. In Fig. 5A, it is observable that droplets are descending with negative acceleration due to the LDEP force generated by the non-uniform electric field from the electrodes. Between electrodes II and III, the electric field remains constant in the sliding direction but alters only in the direction normal to the surface. In this scenario, LDEP force amplifies the hysteresis effect, resembling an adhesive force in vertical direction to the surface due to the electrode configuration. We computed the hysteresis force per unit length from CAH and expressed it as:

3 $$\Delta f=\gamma \iota \nu \times \left(\text{cos}\theta \alpha -\text{cos}\theta r\right)$$

where $\theta \alpha$ is the advancing angle, and $\theta r$ is the receding angle^32–35^. The droplets have a diameter of 3 mm. From the CAH results with all electrodes activated at 200 V, we discovered the hysteresis force to be around − 0.07 mN, slightly surpassing the gravitational force, which is congruent with the findings in Fig. 5A.

On the other hand, the gradient of the electric field induces LDEP force at the edge of the electrode. The electric fields are localized to the surface on account of the fact that interdigitated electrodes exhibit a distinct electric field distribution. Thus, electrodes possess a strong electric field gradient near the surface. In context, LDEP forces act as a notable resisting force in the horizontal direction on the water droplet, as depicted in Fig. 4B. Here, the LDEP force predominantly acts on the droplet, preventing it from leaving the electrode edge. The aggregate force exhibited in Fig. 5A also shows a significant force at the electrode edges. When we subtract the gravitational force from the total force, we obtain the LDEP force. As the droplets traverse off electrode IV, the LDEP force reaches its peak. Consequently, we plotted the LDEP force against different voltages at the 18.75 mm position, as illustrated in Fig. 5B. It is discernible that the LDEP force at 200 V is almost quadruple the gravitational force. The magnitude of LDEP force aligns closely with previous research^23^, which investigated the adhesive force and surface tension during the extraction of water droplets under AC voltages. This comparison shows that our results can be elucidated by the LDEP force.

## Materials and methods

To prepare superhydrophobic interdigitated electrodes surface, indium tin oxide (ITO) glass served as the substrate for fabricating our IDE (the spacing and the width of the IDE are both 50 μm) by laser engraving. First, the interdigitated electrodes underwent the chemical vapor deposition method to deposit a 40 nm-thick insulating layer of SiO2. Subsequently, we applied and spin-coated the nano-particle PTFE solution (60 nl, TeflonTM 30B from The Chemours Company) on the insulating layer. The nano-particle PTFE solution has a particle diameter of 0.22 μm. The coated electrodes then went through a two-stage baking process. In the first stage, electrodes were baked for 20 min at 120 °C to remove moisture. In the second stage, we repeated the spin-coating process, and the coated electrodes underwent a 30-min baking process at 300 °C to establish our superhydrophobic surface. The final 2 layers of Teflon had a thickness of 1.5 μm. The presence of nanoparticle coating contributes to the roughness of our device's surface, enhancing its superhydrophobic properties.

In the experiment of static contact angle measurement, a water droplet was positioned flat on the superhydrophobic interdigitated electrodes surface (5 mm × 10 mm) with a 50 μm spacing of electrodes to accurately determine the contact angles. The contact angle measurements using the Image J software exhibited a 2-degree error. A 10 kHz AC electric field was then applied to the surface to measure the contact angle under the influence of the electric field.

In the experiment of measuring dynamic angle (advancing and receding) and CAH, we prepare a motorized stage equipped with interdigitated electrodes (16 mm × 4.8 mm). LabVIEW software was used to control the platform's sliding at constant speeds.

## Conclusion

We observe a deflection in droplets at the edge of interdigitated electrodes as they slide down under the influence of applied electric fields. Through analyzing the reaction time and the changes in contact angle in response to varying voltages, we deduce that alterations in contact angle are not the principal factors contributing to the deflection of water droplets. Moreover, dynamic contact angles are assessed while in motion. The contact angle hysteresis (CAH) of the droplets is meticulously analyzed, revealing that the variance in CAH between interfaces is not the primary factor contributing to the observed deflection movement of the droplets. Subsequently, we undertake a force analysis of water droplets on inclined surfaces featuring diverse electrode patterns. Through this analysis, we are able to derive both the total force and the hysteresis force, which affirms that the liquid dielectrophoresis (LDEP) force serves as the driving force behind the deflection.

This study introduces a liquid selector predicated on the dielectrophoretic force. The deflection of droplets is achieved on interdigitated electrodes under the application of non-uniform electric fields. This elucidated mechanism holds potential for applications in droplet splitting and transport, enabling exertion of forces during the dynamic process of droplet movement.

### Supplementary Information

Supplementary Video 1.

## Data Availability

The datasets used and/or analysed during the current study available from the corresponding author on reasonable request.
